# Delphi-research exploring essential components and preconditions for case management in people with dementia

**DOI:** 10.1186/1471-2318-10-54

**Published:** 2010-08-09

**Authors:** Paul-Jeroen Verkade, Berno van Meijel, Cindy Brink, Harmieke van Os-Medendorp, Bauke Koekkoek, Anneke L Francke

**Affiliations:** 1 INHolland University for Applied Sciences/Research Group Mental Health Nursing, Amsterdam, The Netherlands; 2 Geriant Mental Health Care, Department for Diagnostics and Casemanagement of Dementia (DOC-team), Heerhugowaard, The Netherlands; 3 Department of Dermatology and Allergology, Faculty Master of Science in Nursing course, University Medical Centre Utrecht, The Netherlands; 4 Altrecht Mental Health Care, Department of Outpatient Community Care, Zeist, The Netherlands; 5 NIVEL - Netherlands institute for health services research, Utrecht, The Netherlands; 6 Department of Public and Occupational Health, EMGO Institute for Health and Care Research (EMGO+) of VU University Medical Center Amsterdam, The Netherlands

## Abstract

**Background:**

Case management programmes for home-dwelling people with dementia and their informal carers exist in multiple forms and shapes. The aim of this research was to identify the essential components of case management for people with dementia as well as the preconditions for an effective delivery of case management services.

**Method:**

The method used to carry out the research was a modified four-phase Delphi design. First, a list of potentially essential components and preconditions for the provision of case management was drawn up on the basis of a literature review and a subsequent focus group interview. The list was then validated by experts in a first Delphi survey round, following which the researchers translated the list items into 75 statements. In the second Delphi survey, the experts rated the statements; in the third Delphi round, they rated 18 statements on which no consensus had been reached in the second round.

**Results:**

The experts were able to build consensus on 61 of the 75 statements. Essential components of case management for people with dementia are: information, support and counselling, coordination of the care provided and, to a lesser extent, practical help. A patient-centred approach was found to be one of the key aspects of providing case management services. Essential preconditions are: vision, care relationship, structured methodology, integration of case management into the health care chain, and the case manager's level of training and expertise.

**Conclusions:**

We recommend that, based on the essential components and preconditions referred to above, quality criteria be developed for the provision of case management for people with dementia. Furthermore, we suggest the conduct of additional research to assess the effectiveness of case management in people with dementia.

## Background

Dementia is a disorder which particularly affects elderly people. The syndrome is characterised by a decline in cognitive function coupled with mood changes as well as changes in behaviour and personality. Dementia is usually irreversible and progressive in nature [1]. Worldwide there are an estimated 24 million people with dementia and this number is expected to double in the next 20 years [2]. The majority of people with dementia are still living at home [3]. Relatives often are highly involved in the care for home-dwelling dementia patients [4], as a result of which dementia has a strong impact on the well-being and functioning not only of the patients themselves, but also of their informal carers. Dementia is the predominant reason for admission of elderly people to a nursing home [5].

Case management is becoming increasingly popular as a strategy for supporting home-dwelling dementia patients and their family. Case management is defined for this purpose as a client-centred strategy to improve the coordination and continuity of the delivery of services, especially for persons with multiple and complex needs [6].

Publications on the effects of case management for people with dementia are limited in number [7-20]. Except for Jansen's thesis [13], all research papers published report significant positive effects of case management strategies. Some evidence was found to suggest a decrease in behavioural and psychological symptoms in demented patients [9,11], a reduction in distress for dementia caregivers [9], a reduction in caregiver burden and depression [17], deferred placement of demented patients in long term institutional care [12] and increased use of community services among demented patients and their caregivers [16]. Strikingly, the studies conducted show great variations in terms of case management model applied and outcome measures used. Consequently, the results of the effectiveness studies are far from univocal and conclusive.

In the studies concerned, the case management programmes for people with dementia take the form of integrated care models [21] showing different combinations of any of the following components: information [7,8,13-18], support and counselling [12-18], organisation and coordination of the care provided by other caregivers [7,8,11,14-18], behaviour management [9,11], training and coaching of the informal carers [9,12,14-18], and crisis interventions and a 24-hour availability of services [12]. Case management can be offered on a multi-disciplinary [9,12] or mono-disciplinary basis [14-18]. The models applied also vary in terms of their organisational structure, with case management sometimes being set up by primary healthcare providers, such as general practitioners or home care services [9], and sometimes by general/university hospitals or mental healthcare institutions [7,8,10].

In conclusion, there is no general consensus at present about the essential components of a case management programme for people with dementia or about the way of organizing those components. This means that case management strategies vary hugely in terms of both content and organization, largely depending on national, regional and local practices as well as random factors. It is impossible to draw any unequivocal conclusions about the effects and quality of case management programmes for people with dementia.

The purpose of this study was to find a consensual expert definition of the essential components of case management for home-dwelling people with dementia and their informal caregivers.

The key questions in the study were as follows:

1. What do experts regard as essential components of case management programmes for people with dementia?

2. Which preconditions are to be fulfilled to provide the essential components of case management?

## Methods

### Study Design

Our aim was to make use of the Delphi method to build consensus about the essential components that should form part of case management programmes for people with dementia and the preconditions needed for an effective implementation of those programmes. The Delphi method involves a structured process of collecting information on a specific subject or problem from a panel of experts through a series of questionnaires. The questionnaires generally consist of both qualitative and quantitative elements [22]. Consensus-building techniques like the Delphi method are useful in situations in which existing literature is incomplete and inconsistent [23]. The study design we used was a Delphi technique in which experts were asked to respond to items of case management identified through literature reviews and a focus group interview with experts [24]. This approach was considered practical because the literature published already suggested a number of potentially suitable components of relevant case management programmes and the interview with the focus group allowed a further exploration of the literature data through discussions among the focus group members [25]. The first Delphi survey round was then used to validate the items concerned [26]; the next rounds were designed to build consensus about the items validated.

### Panel Member Selection

The method used to compose the Delphi panel was a purposive sampling of Dutch experts in the field of case management in people with dementia. The population consisted of experts involved: (1) in the practical use of case management programmes for people with dementia; or (2) in scientific research or policy-making in the field. Forming a mix of these two groups of experts was considered opportune as it allowed the experts to combine practical expertise with the results of available research.

Delphi research does not have a tradition of generally accepted selection criteria, and there is no fixed rule about minimum or maximum numbers of panel members [22]. According to Migchelbrink [27], if the group is homogenous, 10 to 15 participants should suffice to bring to the fore all ideas and opinions on the problems posed. In the present study, the group of experts was homogenous in terms of the area investigated: all of them worked in the field of dementia case management. In terms of composition, however, the group was heterogeneous: some of the experts had a practical background, whilst others had built up experience in the field of research or policy-making. By analogy to Migchelbrink's calculation [27], therefore, 30 participants were considered sufficient to fulfil the requirements of a quality Delphi research design.

The following inclusion criterion was defined for the experts with a practical background: a job as a case manager for people with dementia, or as a nursing home physician, or as a psychiatrist working on a dementia case management team. Eligible participants were selected via the network of researchers, as well as from the report published by Ligthart [28] on case management teams in the Netherlands and through a Google search for Dutch case management teams.

The inclusion criterion for experts with a research or policy background was as follows: publication of one or more research or policy papers about general outpatient care for people with dementia or about specific case management for people with dementia.

The experts were asked by email whether they would be willing to participate in the focus group interview and in the subsequent Delphi survey rounds.

### Data Collection

The study was conducted in four phases spread over the period from December 2008 to April 2009: (1) literature review; (2) focus group interview; (3) first Delphi survey round to validate the pre-selected items; (4) second and third Delphi surveys designed to score items with a view to reaching consensus.

(1) The first phase consisted of a systematic review of literature reporting on the effects of case management on home-dwelling patients with dementia. This review culminated in a preliminary list of components of case management programmes for people with dementia.

(2) The preliminary list of components was discussed during a focus group interview with eight of the experts in order to review the components against the expertise of the experts. The focus group interview was led by the second author (BvM) and was recorded on audiotape. An analysis of the interview materials resulted in an extended and edited list of potentially suitable components of case management programmes for people with dementia.

(3) In the first Delhi survey round, the experts received an email asking whether they missed any components from the list and whether the items included in the list were described to their satisfaction. Their responses were processed and a number of statements were drawn up covering essential components of case management programmes for people with dementia and the preconditions needed for an effective implementation of the programmes.

(4) The second Delphi survey round, also conducted by email, was designed to score the ratings which the experts gave to the various components. The experts were asked to score each statement on a 7-point Likert scale, with the response categories ranging from "totally disagree" (score 1) to "totally agree" (score 7). The experts were also invited to underpin their choices.

(5) In the third Delphi survey round, the experts again received an email, in which they were asked to indicate their agreement/disagreement with a number of statements on which no consensus had been reached in the second Delphi round. The statements in question were accompanied with the mean scores assigned to them in the previous round and the explanations and justifications given by the experts.

### Analysis

The interview with the focus group was written out verbatim on the basis of the tape recording. In a next step, the text was analysed to find new components in addition to the ones distilled from the literature examined. The results were processed into a list of components which was used as a basis for the first Delphi survey, in which the experts validated the list in terms of substantive completeness and textual accuracy. The new list of components was then translated into statements. A log was kept during the process of analysis and the first Delphi round, and the researchers (PJV, BvM, CB) duly discussed all steps taken and choices made during this phase.

The scores from the second and third Delphi surveys were entered into the Statistical Package for the Social Sciences (SPSS) 17.0. For each score, the mean value, the standard deviation, and the modus were calculated. The level of consensus was established based on the standard deviation: the basic assumption was that consensus was reached at a standard deviation level below 1.5 (see Koekkoek *et al*. [26]). Components of case management were considered essential if the mean score for a statement was 5.5 or higher, meaning that the experts had ticked the box "agree" or "totally agree".

The responses scored and explanations given were processed without reference to the originators. All experts were informed of the anonymised results.

## Results

### Survey Responses

Fifty potential experts were approached by email, 35 of whom responded. Five experts did not wish to participate: two of them stated that they did not consider themselves sufficiently qualified to be called experts in the field of case management for people with dementia; the other three were too busy. In the end, thirty experts agreed to participate. Fourteen experts were practising professionals: nine case managers for people with dementia, three team managers, one geriatrician, and one psychiatrist. Thirteen of these experts had been in their position for more than a year. One case manager had been working as a dementia case manager for four months, but had been active as a researcher in the field of dementia before that.

The other sixteen experts were scientific researchers or policy makers in the field of case management for people with dementia. To represent the patients' perspective in our study, a policy adviser of the Dutch Patients Association for dementia patients also participated in the panel of experts.

Invitations for the focus group interview were extended to the first twelve experts who stated that they were willing to be part of the focus group. Two experts cancelled at the last minute due to illness, two others due to an emergency. Therefore, the focus group interview was held with eight experts, five of whom with a practical background, three with a scientific background.

In the first and second Delphi survey rounds, 28 of the 30 experts (93%) responded. In the third round, 29 of the 30 experts (97%) responded. The reason stated for not responding was an overloaded work schedule.

### General Results

Table 1 shows the scores of the experts on the 75 statements set out in the second and third Delphi surveys. The turning point from non-essential to essential was set at a mean score of 5.5 or higher (within the range from 1 to 7). The end result is that the experts built consensus on 61 statements, 44 of which were qualified as essential.

**Table 1:** Scores of Experts on Statements of the Second and Third Delphi Survey Rounds

75 statements	SD<1.5, Mean>5.5			SD<1.5, Mean<5.5			SD>1,5			Total
**Results of 2^nd ^Delphi round**										

Vision and basic assumptions	9			1			3			13

Organisational and operational preconditions	13			1			8			22

Components of case management	21			12			7			40

**Total**	**42**			**15**			**18**			**75**

										

**Results of 3^rd ^Delphi round**										

Vision and basic assumptions	9			2			2			13

Organisational and operational preconditions	14			3			5			22

Components of case management	21			12			7			40

**Total**	**44**			**17**			**14**			**75**

(SD = Standard Deviation, Mean = Mean Score)

The statements were split up into the following categories: vision and basic assumptions (table 2), organisational and operational preconditions (table 3), and components of case management for people with dementia (table 4). Below, the specific results are discussed by category.

**Table 2:** Scores on Statements about Vision and Basic Assumptions

Statements with consensus, rated as essential	N	M1	SD	M2
Case management for people with dementia must be targeted towards both the patient and their system.	28	6.82	0.39	7

A dementia case manager must focus not only on existing problems arising from the disease, but also on preventing potential problems for the patient and their system.	28	6.57	0.50	7

A permanent case manager for each patient is an absolute precondition for the delivery of quality case management.	28	6.25	0.75	7

Case management for people with dementia must be based on the needs and desired of the patient and their system.	28	6.21	0.83	7

A dementia case manager must strive towards empowerment of both the patient and their system.	28	6.21	1.13	7

Case management for people with dementia can only be set up appropriately if it is integrated in the overall care chain.	28	6.18	1.09	7

A provider of case management services for people with dementia cannot perform properly without having developed a clear vision on dementia.	28	6.11	0.88	6

A provider of case management services for people with dementia cannot perform properly without having drawn up a specific set of goals and targets to be achieved.	28	5.89	1.03	6

Dementia case managers who cannot select their care chain partners objectively and independently cannot deliver quality case management.	27	5.67	1.49	6/7

**Statements with consensus, rated as non-essential**	**N**	**M1**	**SD**	**M2**

Dementia case managers cannot provide proper care without the support of a doctor.	28	5.36	1.47	6

Dementia case managers cannot provide proper care without the support of a psychologist.	27	4.63	1.52	5
	28	4.28	1.49	5

**Statements without consensus**	**N**	**M1**	**SD**	**M2**

Diagnostics and the treatment of dementia must form an integral part of a case management programme for dementia patients.	28	4.96	1.80	6
	29	5.07	1.56	6

Case management should also be available for people who are suspected to have dementia (i.e. even before the diagnosis is made).	28	5.29	1.54	6
	29	4.72	2.07	6

(**M1 **= Mean, 1 Totally disagree, 2 Disagree, 3 Disagree slightly, 4 Neutral, 5 Agree slightly, 6 Agree, 7 Totally agree, **SD **= Standard Deviation, **M2 **= Modus)

**Table 3:** Scores on Statements about Organizational and Operational Preconditions

Statements with consensus, rated as essential	N	M1	SD	M2
Dementia case managers may be expected to provide active input in their cooperation with the chain partners.	28	6.54	0.59	7

No proper case management can be delivered without care diagnostics, i.e. assessment of problems, limitations, handicaps, and wishes of the patient and their system.	28	6.54	0.64	7

No proper case management can be delivered without keeping a care file.	28	6.43	0.69	7

No proper case management can be delivered without firm agreements with the chain partners about coordination, exchange of information and caregiving responsibilities.	28	6.36	0.91	7

A dementia case manager is responsible for setting up, maintaining and concluding a manageable care relationship with each patient and their system.	27	6.04	1.13	6/7

A higher professional qualification (HBO) is the minimum level of education required for a dementia case manager to be able to perform her duties properly.	28	5.96	1.26	7

A dementia case manager must work in such a transparent manner as to facilitate her immediate replacement in case of absence due to a period of leave or illness.	28	5.89	1.32	6

A dementia case manager must make house calls.	28	5.82	1.09	6

A dementia case manager must make use of protocol- and evidence-based interventions where possible.	28	5.64	1.52	6
	29	5.66	1.26	6

The use of standardised measuring instruments for diagnostic purposes and for monitoring patients is essential to the delivery of quality case management.	28	5.57	1.03	6

A good dementia case manager must discuss her own job performance and the existing care relationship with each patient and her system at least once a year.	27	5.56	1.45	6

The use of standardised measuring instruments for diagnostic purposes and for monitoring the informal carers is essential to the delivery of quality case management.	27	5.52	1.28	6

High-quality case management for people with dementia cannot be achieved if case management is not implemented methodically.	26	5.50	1.27	6

A dementia case manager must always be reachable by telephone during office hours to answer questions and/or respond to emergencies.	28	5.50	1.29	6

**Statements with consensus, rated as non-essential**	**N**	**M1**	**SD**	**M2**

Dementia case managers can only perform their duties properly if they keep up with relevant professional literature.	27	5.37	1.31	6

A good dementia case manager should perform and discuss yearly multi-disciplinary care evaluations with the patient and their system.	27	5.52	1.58	6
	28	5.36	1.47	6

A dementia case manager must attend a minimum of six peer consultation meetings per annum in order to assure the quality of the case management delivered.	28	5.07	1.56	6
	29	4.52	1.46	5/6

**Statements without consensus**	**N**	**M1**	**SD**	**M2**

A dementia case manager must prepare a personal care plan for the patient and their system within two months.	28	5.29	1.72	6
	26	4.73	1.76	6

Good case management requires a 24/7 on-call service for emergency situations.	26	5.00	1.63	6
	29	4.55	2.03	6

Dementia case managers cannot properly perform their duties without a nursing education.	26	4.65	1.65	6
	29	4.03	1.88	3

An ideal case load for dementia case managers involves 12 to 15 patients per 8 hours of work.	24	4.46	1.62	6
	28	3.96	1.50	4

A case manager should have structural contacts with patients and their systems at least once every six weeks, even if there is no demand for care.	28	4.64	1.73	6
	28	3.75	1.80	6

(**M1 **= Mean, 1 Totally disagree, 2 Disagree, 3 Disagree slightly, 4 Neutral, 5 Agree slightly, 6 Agree, 7 Totally agree, **SD **= Standard Deviation, **M2 **= Modus)

**Table 4:** Scores on Statements about Components of Case Management for People with Dementia

Statements with consensus, rated as essential	N	M1	SD	M2
A good dementia case manager must be sensitive to the differences in perception between patients and their informal carers.	28	6.64	0.49	7

Care coordination is one of the dementia case manager's key duties.	28	6.61	0.69	7

Good case management requires a systematic listing - and discussion with the patient and their system - of the specific needs for care and support that can be provided.	28	6.54	0.51	7

A dementia case manager can only provide proper support and counselling if there is room for addressing the emotions of both the patient and their system.	28	6.54	0.51	7

Informing patients and their systems about the disease and the care and support options that are available is one of the dementia case manager's key duties.	28	6.54	0.84	7

A dementia case manager must give the patient and their system the confidence that they can jointly work on finding solutions to the problems caused by dementia.	28	6.50	0.58	7

Offering emotional support and counselling to patients and their systems is one of the dementia case manager's key duties.	28	6.43	0.74	7

Coaching the system on how to deal with behavioural changes in the patient is one of the dementia case manager's key duties.	27	6.41	0.64	7

The provision of information will have no effect if the information is not fine-tuned to the patient and their system.	27	6.41	0.80	7

A good dementia case manager will help patients and their systems to make difficult decisions.	28	6.29	0.66	6

A dementia case manager cannot function without establishing a sense of 'being there' for all persons concerned or without showing dedication and commitment.	28	6.29	1.049	7

A good dementia case manager actively provides information to the patient and their system.	27	6.22	0.85	6

A good dementia case manager will prepare patients and their systems for the effects and implications of the disease.	26	6.12	0.77	6

A dementia case manager should protect the interests of the patient and their system.	28	6.04	0.84	6

A dementia case manager should systematically consult with other care providers involved with the patient.	28	6.00	0.94	6

A good dementia case manager will evaluate and monitor the quality of the care provided by third parties, and will intervene whenever necessary.	26	5.85	1.41	7

A good dementia case manager will facilitate a safe environment for patients living at home, e.g. by applying for technical aids or home adjustments or by facilitating interventions in cases of danger.	27	5.78	1.12	6

A good dementia case manager will reinforce the patient's social system and promote the patient's involvement in community life.	28	5.75	0.93	6

Offering help with filling in forms (e.g. applications for home care or household help) is one of the dementia case manager's key duties.	27	5.70	1.27	6

Compassionate interference in cases where patients refuse the care they need is one of the dementia case manager's key duties.	27	5.70	1.27	6

A good dementia case manager is active in case finding, e.g. by offering consultations to fellow care providers and/or through public information.	28	5.68	1.09	6

**Statements with consensus, rated as non-essential**	**N**	**M1**	**SD**	**M2**

A good dementia case manager schedules separate meetings with the patient's system.	28	5.43	1.07	6

A case manager must always share her information with the patient in several meetings.	27	5.41	1.01	6

A good dementia case manager will inform the patient and their system of the diagnosis, the current status, and future prospects.	28	5.39	1.40	6

A good dementia case manager will organise a group discussion with the patient's system and the caregivers involved in order to coordinate the care.	28	5.32	1.16	6

A dementia case manager should discuss all information originating from other care providers with the patient and their system.	27	5.19	1.30	6

Participating in educating the public about dementia, e.g. by participating in informal meetings in 'Alzheimer Cafes', dementia information centres or dementia shops, is one of the case manager's key duties.	28	5.18	1.31	6

Organising consultation, coaching or training sessions with other professionals, e.g. about behavioural changes, is one of the dementia case manager's key duties.	28	5.04	1.40	6

A dementia case manager may be expected to counsel support groups for informal carers of people with dementia.	28	5.00	1.41	4

A dementia case manager can only provide information effectively by offering the information to the patient and their system both orally and in writing.	28	4.71	1.49	4

A dementia case manager should be able to lead discussion groups organised for people with dementia.	28	4.71	1.49	6

A good dementia case manager will invite the patient and their system to visit assisted living facilities.	28	4.68	1.34	5

Giving training or educational courses to informal carers is one of the case manager's key duties.	27	4.30	1.44	4

**Statements without consensus**	**N**	**M1**	**SD**	**M2**

A good dementia case manager also offers assistance to informal carers after the patient is admitted or has died.	28	5.39	1.58	6
	29	5.34	1.70	6

A dementia case manager should be able to handle elements of a patient's treatment in the context of a multi-disciplinary treatment plan.	28	5.25	1.78	6
	28	4.89	1.89	6

Case manager and patients must communicate on a level playing field when sharing information, making choices about the care provided, etc.	27	5.00	1.64	6
	27	4.48	1.67	4

A dementia case manager should organise transport for patients and their systems to training centres, day activity centres, appointments with professional carers, etc..	28	4.39	1.52	5/6
	24	4.04	1.73	2/5

A case manager is not a specialist and can at most support or counsel, but not treat, a patient.	28	4.07	1.68	4
	28	3.64	1.52	3

A dementia case manager can give treatment independently.	28	3.79	1.89	6
	29	3.45	1.59	2

A dementia case manager should be willing to provide occasional practical help, e.g. by performing a household chore or running an errand.	26	3.62	1.63	2
	29	3.07	1.51	2

(**M1 **= Mean, 1 Totally disagree, 2 Disagree, 3 Disagree slightly, 4 Neutral, 5 Agree slightly, 6 Agree, 7 Totally agree, **SD **= Standard Deviation, **M2 **= Modus)

### Vision and Basic Assumptions

In the category headed Vision and Basic Assumptions, the experts qualified 9 of the 13 statements as essential (see table 2). The statements mainly concerned issues pertaining to the approach towards patients and their systems, and the cooperation with other caregivers, the 'chain partners'.

One of the approach elements considered essential in case management was the focus on both patient and informal carer, and on the individual needs of these persons. Also essential were the tasks of dementia case managers: (i) to promote empowerment in the informal carers and/or the patient; and (ii) with a view to prevention, to be alert to signs of potential problems. Finally, the experts highlighted the importance of having a named case manager for a patient throughout the whole care process.

As concerns the cooperation with chain partners, the experts agreed that case management in people with dementia was impossible unless it was integrated into the overall care chain. In other words, case management would only be effective if the professional care providers of the various organisations were prepared to work together and to recognise the case manager as the coordinator of all care.

The experts qualified two statements in this category as non-essential. The statements in question touched on the question of whether a physician's or a psychiatrist's direct involvement was critical to an effective implementation of case management programmes. In the explanations, several experts did stress the usefulness of physicians and psychologists, but added that they were not convinced of the actual need of their involvement for the effectiveness of case management.

The experts failed to reach consensus on two of the statements. The first of these statements was the question of whether case management for people with dementia should be available even before the diagnosis was made. Opponents argued that case management at such an early stage was too expensive and that it was the general practitioner's responsibility to provide care at that stage. Advocates contended that patients and their systems went through a stage of great uncertainty at the beginning of the illness, and would benefit enormously from the support which case managers could give.

The second statement in question was whether dementia diagnostics and treatment should form an integral part of case management programmes for people with dementia. Opponents believed that diagnostics and treatment were fields of medical expertise and should not form part of a case manager's portfolio. Advocates referred to the importance of multi-disciplinary cooperation in diagnostics and treatment and the advantages of a case manager's active participation in the process.

### Organisational and Operational Preconditions

In the category headed Organisational and Operational Preconditions, the experts qualified 14 of the 22 statements as essential (see table 3). The statements can be divided into the following four main subgroups:

(1) The need to set up and maintain a good relationship of care, e.g. by making house calls, being reachable by phone, and holding a yearly - or more frequent - case management assessment interview with the patient and their informal carer or carers.

(2) A systematic method of working, through structured care diagnostics supported by appropriate measuring instruments and properly updated files that are transparent to fellow case managers in emergency situations.

(3) Active input by case managers in their cooperation with other intra- and extramural chain partners, and firm agreements about shared responsibilities.

(4) Evidence of the case manager's professional status: minimum higher professional qualification and maximum use of evidence-based interventions.

Three statements in this category were considered non-essential. Two of these concerned the case manager's level of expertise and practical skills: the experts did not consider the process of keeping up with relevant professional literature and attending a minimum of six peer consultation meetings per annum to be essential. Nor was a minimum yearly multi-disciplinary care evaluation regarded as essential.

There were five statements on which no consensus was reached. For example, the experts disagreed about what was the ideal practicable case load. Some experts argued that a manager's case load depended on several factors, including the moment of the case manager's first involvement in the process (prior or after the diagnosis), the extent of the care required, and the complexity of the patient's systems. They also were unable to reach consensus about the preparatory *nursing *education of case managers that was suggested. One of the arguments speaking against this proposition was that a case manager with a degree in social work, for example, could also make a valuable contribution by focusing specifically on the patient's social context. Conversely, some experts argued that no integral approach towards complex somatic, psychiatric, and social issues could be achieved without a specific nursing background. As concerns the other non-consensual statements, reference is made to table 3.

### Components of Case Management for People with Dementia

In the category headed Components of Case Management for People with Dementia, the experts qualified 21 of the 40 statements as essential (see table 4). The components considered essential can be grouped as follows:

(1) Information of the patients and their systems;

(2) Support to the patients and their systems;

(3) Coordination and monitoring of the care provided by others;

(4) To a lesser extent: provision of practical help.

The support component was subdivided into specific aspects of support, such as emotional support, counselling, and coaching of informal carers on how to deal with changes in the patient's behaviour. According to the experts, it was essential for case managers to protect the interests of the patients and to prepare patients and their systems for accepting and dealing with the implications of dementia. Another component identified as essential was 'compassionate interference': the unsolicited provision of care to people with dementia who need to receive care. Examples of essential practical help are the provision of assistance in creating a safe home situation and filling in forms. A number of statements specifically referred to the provision of these case management components by describing attitudinal aspects of a case manager, such as 'a good dementia case manager must be sensitive to the differences in perception between patients and their informal carers' or 'a case must manager must give the patients and their systems confidence' or 'a dementia case manager cannot function without establishing a sense of *being there *for all persons concerned'.

The experts considered twelve statements in this category to be non-essential to case management. Five statements concerned matters such as coaching informal carers in support groups, leading discussion groups for patients with dementia and/or informal carers, and participating in public information and education. Other statements which were qualified as non-essential mainly concerned specific methods of providing information and support, such as the conduct of separate interviews with the patient's system or the provision of information to patients on a number of occasions.

There were seven statements in this category on which the experts could not reach consensus. Three of these statements had reference to the question of whether a case manager could treat or perform a role in the treatment of dementia. A number of experts referred to the lack of clarity of the definition of 'treatment' and, for that reason, felt it difficult to rate the statements in question. Arguments speaking against the statements were that the treatment of patients primarily fell within the domains of medicine and psychology. On the other hand, it was argued that case managers were, in fact, specialists who were well trained in treatment strategies and techniques.

## Discussion

The aim of the present study was to define the essential components and essential preconditions of case management for people with dementia. To achieve this aim, a four-phase Delphi design was used in which 75 statements were drawn up and then evaluated by a panel of experts. The experts rated 44 statements as essential and 17 as non-essential. There were 14 statements on which no consensus could be reached, but none on which the experts produced a mean score below Neutral, which indicates that the experts did not disagree with any of the statements. A possible explanation for this score is that all components were validated by the experts in the first Delphi survey round, so that they viewed all components listed as valid components of case management for people with dementia.

Essential components of dementia case management are: (1) information of the patients and their systems; (2) support to the patients and their systems; (3) coordination and monitoring of the care provided by others; (4) to a lesser extent: provision of practical help.

As concerns the preconditions for effective case management, a distinction is to be made between preconditions pertaining to vision, on the one hand, and organisational and operational preconditions, on the other. The preconditions pertaining to vision mainly have reference to the question of how patients and their family should be approached. The appropriate way of offering case management is to follow a patient-centred strategy, offering case management based on the needs and wishes of the patient combined with a focus on both the patient and the informal carers. Successful case management thus requires that case managers be able to rely on a shared case management vision to give direction to the day-to-day care provided in practice.

Organisational and operational preconditions reveal themselves in: (1) a methodical approach; (2) the need to set up and maintain a good care relationship; (3) the case manager's role in cooperating with the chain partners; and (4) the case manager's level of training and expertise. These preconditions are important because they directly impact the practical implementation of the dementia case manager's job.

There are several studies which address the effects that case management has on people suffering from dementia [7-20], but these studies contain virtually no detailed information or justification pertaining to the nature or scope of the intervention. This present study offers an answer, to some extent at least, as it makes explicit how case management should be built up in terms of both substance and form.

In each of the categories, there are several statements on which no consensus could be reached. For example, in the category headed 'Vision and Basic Assumptions', the experts were unable to agree on whether or not case management should also be available for people who are suspected to have dementia (i.e. even before the diagnosis is made) and on whether diagnostics and the treatment of dementia must form an integral part of a case management programme for dementia patients. The differences of opinion about the point in time at which case management should start and about the role of diagnostics and treatment within the overall process may also explain the lack of consensus on several statements from the other categories, such as the statement on maximum case load ("An ideal case load for dementia case managers involves 12 to 15 patients per 8 hours of work"). Indeed, the case load per patient and, as a consequence, a case manager's maximum case load depends not only on a patient's demand for care and the scope of the management activities involved, but also on the point in time at which case management begins. And the statement reading "[a] good dementia case manager also offers assistance to informal carers after the patient is admitted or has died" is, in fact, a statement about the point in time at which case management ends and, as a consequence, has an impact on the maximum practicable case load.

In a number of previous studies, whilst the size of the case load was made explicit [10,12,14-20], no explanation was offered for that size, and since none of the studies concerned actually worked out the concept of case management as an intervention, no conclusions could be drawn as regards the reasons for choosing a specific case load. In our study, the stated numbers of patients that made up an average full-time case load varied considerably. The experts explained that the size depended on the level of care required by patients and their care providers, and the start of the care provision in the dementia process. Patients in the early stages of dementia needed a different level of care than those whose dementia had progressed.

The lack of consensus among experts about the question of whether diagnostics and treatment should form an integral part of case management becomes even more evident in later statements, i.e. "[a] dementia case manager should be able to handle elements of a patient's treatment in the context of a multi-disciplinary treatment plan" and "[a] dementia case manager can give treatment independently. A case manager is not a specialist and can at most support or counsel, but not treat, a patient."

The practical implication of this lack of consensus is that there are two different views on service delivery. Firstly, there is the view that case management should start after the diagnostic process has been completed: the case manager offers support, but treatment is given by other service providers. Secondly, there is the view that case management starts even before the diagnosis is made: in this approach, both diagnostics and treatment are integrated into case management. This latter type of case management obviously demands of the case manager an enhanced level of expertise and skills in the field of diagnostics and treatment.

### Diagram

The findings above are illustrated schematically as follows (Additional file 1). The essential components are placed in the centre and are surrounded by the essential preconditions. Additional components on which the experts reached consensus but agreed that they were non-essential (mean score below 5.5) are farther removed from the centre. All this results in a diagram in which a core box of essential case management components is set apart from a broader box of additional components, such as the multi-disciplinary support structure and preventative tasks.

The strengths of the research are its design and the high response rate. The advantage of the Delphi design used was that the experts validated items identified in the literature by means of a focus group interview and an initial Delphi survey round. This made it possible to present the subject-matter in a broad and multi-faceted context. The purposive sampling of field experts and scientific experts was conducive to presenting a broad perspective of case management. In each of the three Delphi surveys, the response rate was 93% or higher. This is a very successful rate which contributed to the reliability and validity of the research. According to Hasson [22] the minimum response rate required for drawing reliable and valid conclusions is 70% in each Delphi round.

The study was conducted with the aid of Dutch experts. To what extent the results can be generalized to other countries depends on the comparativeness of the health care systems concerned and the specific cultural and social aspects of each country. The Dutch health care system is very similar to the systems prevailing in other Western countries, so that the results of the study may well be valid in those countries as well. It would be desirable to repeat the study in an international context.

## Conclusions

To our knowledge, this is the first study in which essential components of case management for people with dementia and the preconditions for effectively providing such case management were assessed.

Essential components of case management for people with dementia are: information, support and counselling, coordination of care and, to a lesser extent, practical help. Essential preconditions can be divided into: (1) preconditions pertaining to vision on care and case management, mainly having reference to the question of how patients and their family should be approached and ultimately transformed into a patient-centred strategy; and (2) organisational and operational preconditions differentiated in terms of care relationship, structural methodology, integration into the health care chain, and training and expertise.

One of the recommendations from the research is that professionals, managers, policy-makers and financiers should be aware of the various forms of case management that exist for people with dementia and of the various ways in which case management can be implemented in practice. In addition, there are a number of specific preconditions to be fulfilled in order to provide for effective case management services. Building awareness is necessary in order to be able to present a joint definition of the requirements to be met in creating case management programmes. This research may form a basis for relevant discussions.

The next step would be to use the essential components and preconditions as a basis for developing minimum quality criteria for case management in people with dementia. Application of quality criteria will reduce (undesirable) differences in case management and enhance the quality of the care provided to people with dementia. Then, there is an urgent need for further international multi-centre research into the effectiveness of case management programmes for people with dementia. In any such research, existing case management services should be standardised based on the quality criteria defined, and uniform outcome measures should be used to assess the effectiveness of the services provided.

## Competing interests

The authors declare that they have no competing interests.

## Authors' contributions

PJV, BvM, HvOM and BK contributed to the development, conceptualization and design of the study. PJV and BvM were responsible for the focus group interview and the data collection (three Delphi survey rounds). PJV and CB were responsible for data transcription, analysis, and preparation of the manuscript. BvM and AF supervised the research process data and provided assistance with data analyses and editing the final manuscript. All authors contributed to the interpretation of the results, and all authors reviewed and approved the final manuscript.

## Pre-publication history

The pre-publication history for this paper can be accessed here:

http://www.biomedcentral.com/1471-2318/10/54/prepub

## Supplementary Material

Additional file 1 **Schematics of case management for people with dementia**. Click here for file

## References

[B1] Ritchie K Lovestone S The dementias Lancet 2002 360 9347 1759 1766 10.1016/S0140-6736(02)11667-9 12480441

[B2] Qiu C De Ronchi D Fratiglioni L The epidemiology of the dementias: an update Curr Opin Psychiatry 2007 20 4 380 385 10.1097/YCO.0b013e32816ebc7b 17551353

[B3] Gezondheidsraad Dementie [Dementia] 2002 http://www.gezondheidsraad.nl/sites/default/files/02@04NR.PDF

[B4] Papastavrou E Kalokerinou A Papacostas SS Tsangari H Sourtzi P Caring for a relative with dementia: family caregiver burden J Adv Nurs 2007 58 5 446 457 10.1111/j.1365-2648.2007.04250.x 17442030

[B5] Berr C Wancata J Ritchie K Prevalence of dementia in the elderly in Europe Eur Neuropsychopharmacol 2005 15 4 463 471 10.1016/j.euroneuro.2005.04.003 15955676

[B6] Hesse M Vanderplasschen W Rapp RC Broekaert E Fridell M Case management for persons with substance use disorders Cochrane Database Syst Rev 2007 4 CD006265 17943902 10.1002/14651858.CD006265.pub2

[B7] Aupperle PM Coyne AC Primary vs subspecialty care: a structured follow-up of dementia patients and their caregivers Am J Geriatr Psychiatry 2000 8 2 167 170 10804078

[B8] Aupperle PM MacPhee ER Coyne AC Blume J Sanchez B Health service utilization by Alzheimer's disease patients: a 2-year follow-up of primary versus subspecialty care J Geriatr Psychiatry Neurol 2003 16 1 15 17 12641367 10.1177/0891988702250507

[B9] Callahan CM Boustani MA Unverzagt FW Austrom MG Damush TM Perkins AJ Fultz BA Hui SL Counsell SR Hendrie HC Effectiveness of collaborative care for older adults with Alzheimer disease in primary care: a randomized controlled trial JAMA 2006 295 18 2148 2157 10.1001/jama.295.18.2148 16684985

[B10] Challis D von Abendorff R Brown P Chesterman J Hughes J Care management, dementia care and specialist mental health services: an evaluation Int J Geriatr Psychiatry 2002 17 4 315 325 10.1002/gps.595 11994884

[B11] Chu P Edwards J Levin R Thomson J The use of clinical case management for early stage Alzheimer's patients and their families American Journal of Alzheimer's Disease 2000 15 5 284 290 10.1177/153331750001500506

[B12] Eloniemi-Sulkava U Notkola IL Hentinen M Kivela SL Sivenius J Sulkava R Effects of supporting community-living demented patients and their caregivers: a randomized trial J Am Geriatr Soc 2001 49 10 1282 1287 10.1046/j.1532-5415.2001.49255.x 11890485

[B13] Jansen APD Effectiveness of case management among older adults with dementia symptoms and their informal caregivers PhD 2007 Vrije Universiteit Amsterdam, Amsterdam

[B14] Miller R Newcomer R Fox P Effects of the Medicare Alzheimer's Disease Demonstration on nursing home entry Health Serv Res 1999 34 3 691 714 10445898 PMC1089033

[B15] Newcomer R Miller R Clay T Fox P Effects of the Medicare Alzheimer's disease demonstration on Medicare expenditures Health Care Financ Rev 1999 20 4 45 65 11482124 PMC4194605

[B16] Newcomer R Spitalny M Fox P Yordi C Effects of the Medicare Alzheimer's Disease Demonstration on the use of community-based services Health Serv Res 1999 34 3 645 667 10445896 PMC1089031

[B17] Newcomer R Yordi C DuNah R Fox P Wilkinson A Effects of the Medicare Alzheimer's Disease Demonstration on caregiver burden and depression Health Serv Res 1999 34 3 669 689 10445897 PMC1089032

[B18] Shelton P Schraeder C Dworak D Fraser C Sager MA Caregivers' utilization of health services: results from the Medicare Alzheimer's Disease Demonstration, Illinois site J Am Geriatr Soc 2001 49 12 1600 1605 10.1111/j.1532-5415.2001.49267.x 11843991

[B19] Vickrey BG Mittman BS Connor KI Pearson ML Della Penna RD Ganiats TG Demonte RW Chodosh J Cui X Vassar S Duan N Lee M The effect of a disease management intervention on quality and outcomes of dementia care: a randomized, controlled trial Ann Intern Med 2006 145 10 713 726 17116916 10.7326/0003-4819-145-10-200611210-00004

[B20] Yordi C DuNah R Bostrom A Fox P Wilkinson A Newcomer R Caregiver supports: outcomes from the Medicare Alzheimer's disease demonstration Health Care Financ Rev 1997 19 2 97 117 10345408 PMC4194475

[B21] Minkman MM Ligthart SA Huijsman R Integrated dementia care in The Netherlands: a multiple case study of case management programmes Health Soc Care Community 2009 17 5 485 494 10.1111/j.1365-2524.2009.00850.x 19694030

[B22] Keeney S Hasson F McKenna H Consulting the oracle: ten lessons from using the Delphi technique in nursing research J Adv Nurs 2006 53 2 205 212 10.1111/j.1365-2648.2006.03716.x 16422719

[B23] Hasson F Keeney S McKenna H Research guidelines for the Delphi survey technique J Adv Nurs 2000 32 4 1008 1015 10.1046/j.1365-2648.2000.01567.x 11095242

[B24] McKenna HP The Delphi technique: a worthwhile research approach for nursing? J Adv Nurs 1994 19 6 1221 1225 10.1111/j.1365-2648.1994.tb01207.x 7930104

[B25] Holloway I Wheeler S Qualitive research in nursing 2002 Oxford: Blackwell Publishing

[B26] Koekkoek B van Meijel B Schene A Hutschemaekers G Clinical problems in the long-term care of patients with chronic depression J Adv Nurs 2008 62 6 689 697 10.1111/j.1365-2648.2008.04645.x 18503653

[B27] Migchelbrink F Praktijkgericht onderzoek in zorg en welzijn [Practice based research in health care and social work] 2007 Amsterdam: Uitgeverij SWP

[B28] Ligthart SA Casemanagement bij dementie [Case management in dementia 2006 http://www.vilans.nl/Site_LDP/docs/PDF/Rapport%20Casemanagement%20bij%20Dementie%20LDP.pdf

