# Supplementary figures and images for: Delphi-research exploring essential components and preconditions for case management in people with dementia
Source: BMC Geriatr. 2010 Aug 9;10:54. doi: 10.1186/1471-2318-10-54 (PMC2928241; doi:10.1186/1471-2318-10-54)

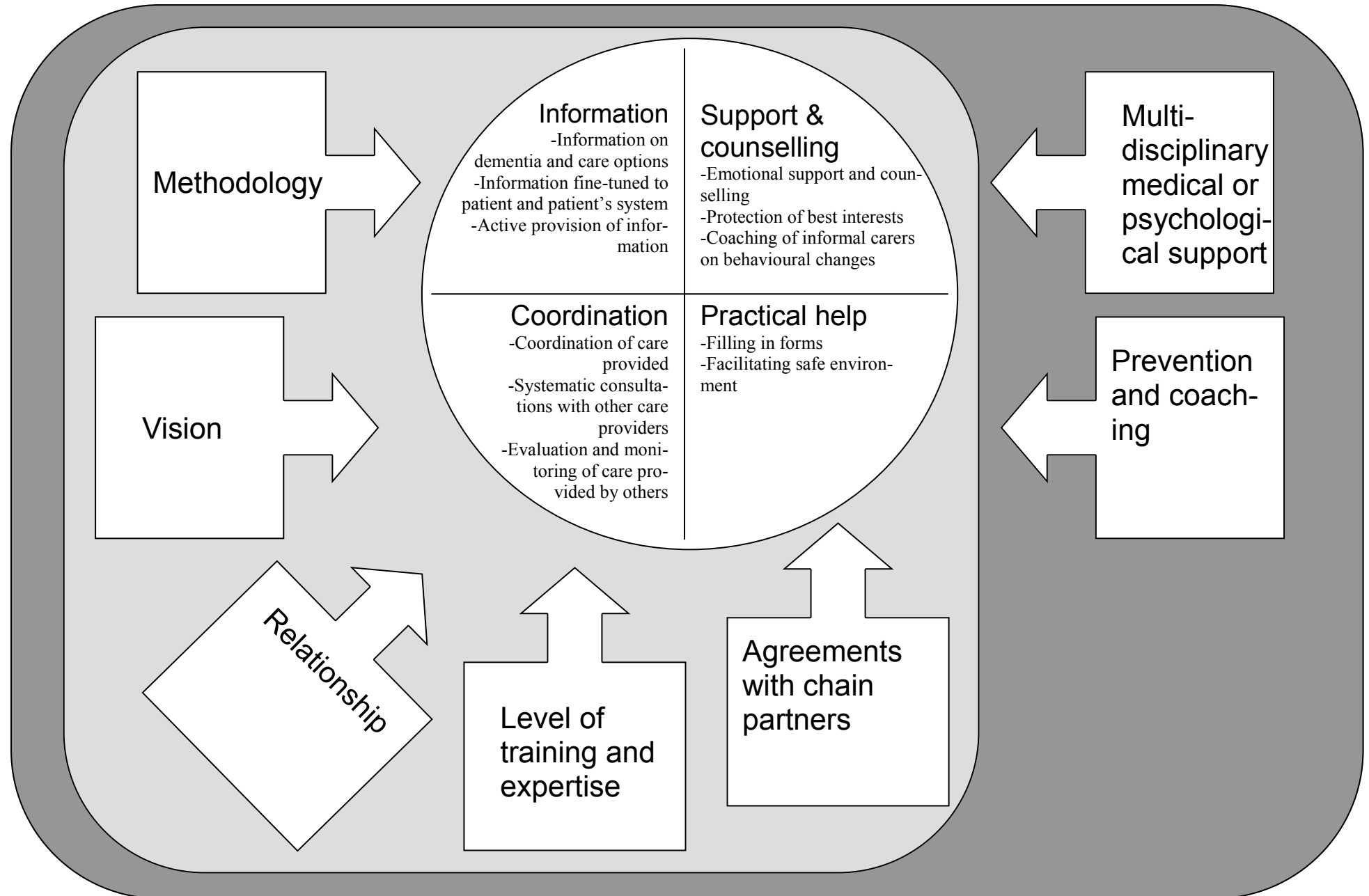

Supplement: Additional file 1 — Schematics of case management for people with dementia. [file 1471-2318-10-54-S1.PDF]
